# Diurnal regulation of RNA polymerase III transcription is under the control of both the feeding–fasting response and the circadian clock

**DOI:** 10.1101/gr.217521.116

**Published:** 2017-06

**Authors:** François Mange, Viviane Praz, Eugenia Migliavacca, Ian M. Willis, Frédéric Schütz, Nouria Hernandez, the CycliX Consortium

**Affiliations:** 1 Center for Integrative Genomics, Faculty of Biology and Medicine, University of Lausanne, 1015 Lausanne, Switzerland;; 2 Swiss Institute of Bioinformatics, 1015 Lausanne, Switzerland;; 3 Department of Biochemistry, Albert Einstein College of Medicine, Bronx, New York 10461, USA;; 4 Bioinformatics Core Facility, Swiss Institute of Bioinformatics, 1015 Lausanne, Switzerland; 7 Center for Integrative Genomics, Faculty of Biology and Medicine, University of Lausanne, 1015 Lausanne, Switzerland; 8 Swiss Institute of Bioinformatics, 1015 Lausanne, Switzerland; 9 Bioinformatics Core Facility, Swiss Institute of Bioinformatics, 1015 Lausanne, Switzerland; 10 Department of Oncology and Ludwig Center for Cancer Research, Faculty of Biology and Medicine, University of Lausanne, 1011 Lausanne, Switzerland; 11 Interfaculty Institute of Bioengineering, School of Life Sciences, Ecole polytechnique Fédérale de Lausanne, 1015 Lausanne, Switzerland; 12 Vital IT, Swiss Institute of Bioinformatics, 1015 Lausanne, Switzerland; 13 Bioinformatics and Biostatistics Core Facility, School of Life Sciences, Ecole polytechnique Fédérale de Lausanne, 1015 Lausanne, Switzerland; 14 Department of Molecular Biology, Faculty of Sciences, University of Geneva, 1211 Geneva, Switzerland

## Abstract

RNA polymerase III (Pol III) synthesizes short noncoding RNAs, many of which are essential for translation. Accordingly, Pol III activity is tightly regulated with cell growth and proliferation by factors such as MYC, RB1, TRP53, and MAF1. MAF1 is a repressor of Pol III transcription whose activity is controlled by phosphorylation; in particular, it is inactivated through phosphorylation by the TORC1 kinase complex, a sensor of nutrient availability. Pol III regulation is thus sensitive to environmental cues, yet a diurnal profile of Pol III transcription activity is so far lacking. Here, we first use gene expression arrays to measure mRNA accumulation during the diurnal cycle in the livers of (1) wild-type mice, (2) arrhythmic *Arntl* knockout mice, (3) mice fed at regular intervals during both night and day, and (4) mice lacking the *Maf1* gene, and so provide a comprehensive view of the changes in cyclic mRNA accumulation occurring in these different systems. We then show that Pol III occupancy of its target genes rises before the onset of the night, stays high during the night, when mice normally ingest food and when translation is known to be increased, and decreases in daytime. Whereas higher Pol III occupancy during the night reflects a MAF1-dependent response to feeding, the rise of Pol III occupancy before the onset of the night reflects a circadian clock-dependent response. Thus, Pol III transcription during the diurnal cycle is regulated both in response to nutrients and by the circadian clock, which allows anticipatory Pol III transcription.

Physiological processes and behavior are subjected to rhythms generated by a circadian timing system that coordinates them with cycles of day and night and allows the anticipation of daily changes (Bass and Takahashi 2010; Dibner and Schibler 2015). At the core of this timing system is a cellular circadian clock, which consists of interconnected transcriptional and translational feedback loops (Cermakian and Sassone-Corsi 2000; Lowrey et al. 2000; Reppert and Weaver 2002; Kim et al. 2014; Partch et al. 2014). The main drivers of the clock are the transcription factors CLOCK and ARNTL (also known as BMAL1), which activate transcription of clock-controlled genes (CCGs) by binding as heterodimers to E-boxes located within their promoters (Gachon et al. 2006; Ukai-Tadenuma et al. 2008; Koike et al. 2012; Crane and Young 2014). Among the CCGs, activated transcription of the *Per* and *Cry* gene families leads to the gradual accumulation of PER and CRY proteins; when these proteins reach a certain level, they heterodimerize and translocate into the nucleus, where they inactivate the CLOCK/ARNTL dimer and thereby repress transcription of their own genes. The degradation of PER and CRY proteins then allows CLOCK/ARNTL-dependent transcription to resume, resulting in a self-perpetuating feedback loop (Asher and Sassone-Corsi 2015). The loop is further stabilized by the ROR and NR1D1/2 proteins, which respectively activate and repress *Arntl* transcription (Preitner et al. 2002), and is fine-tuned by post-translational modifications (Lowrey and Takahashi 2000; Asher et al. 2008).

At the organismal level, the cellular clocks are hierarchically organized. The clocks located in the approximately 20,000 neurons of the suprachiasmatic nucleus (SCN) in the ventral hypothalamus tick in concert and constitute an autonomous, central pacemaker, which can be reset by a photic input pathway from the retina (Ralph et al. 1990). This central pacemaker in turn entrains clocks in peripheral organs through a variety of neuronal and humoral outputs (Yamazaki et al. 2011; Schibler et al. 2016). However, peripheral clocks are also controlled by other clues; notably, the liver clock can be entrained by the nutrient response (Damiola et al. 2000; Dibner et al. 2010; Yamazaki et al. 2011). Thus, in animals subjected to a feeding schedule that differs from normal feeding conditions, such as daytime feeding for mice, the liver clock, but not the SCN clock, slowly shifts to a new phase determined by the imposed feeding schedule (Damiola et al. 2000; Stokkan et al. 2001; Le Minh et al. 2002; Panda et al. 2002).

The circadian clock imposes rhythmic transcription on a large set of CCGs; these rhythmic gene sets differ from tissue to tissue (Zhang et al. 2014), consistent with tissue-specific transcription factors cooperating with core clock transcription factors, such a CLOCK/ARNTL, to direct different transcription programs. However, rhythmic gene expression is not necessarily imposed at the level of transcription; in the mouse liver, rhythmic transcription can give rise to flat mRNA levels, and constant transcription can give rise to rhythmic mRNAs (Koike et al. 2012; Le Martelot et al. 2012). Further, rhythmic proteins can arise from nonrhythmic mRNAs, for example, through rhythmic translation (Reddy et al. 2006; Mauvoisin et al. 2014; Atger et al. 2015; Janich et al. 2015). Global translation is in fact rhythmic in mice, as the fraction of ribosomes in polysomes is slightly elevated during waking hours and decreases in the middle of the day. This results, at least in part, from rhythmic expression and activation of components of the translation initiation complex and the ribosome machinery (Jouffe et al. 2013), produced from RNA polymerase II (Pol II)-transcribed genes for the protein components and from Pol I genes for the large ribosomal RNA components.

The translation machinery is not only comprised of Pol II and Pol I products, but also of the 5S RNA component of ribosomes and of tRNAs, all produced by Pol III. Pol III further contributes to translation by synthesizing RNA molecules involved in the maturation of components of the translation machinery, for example *Rpph1* RNA (RNase P RNA), required for maturation of pre-tRNA, and *Rmrp* RNA (MRP RNA), required for maturation of the large ribosomal RNAs (Dumay-Odelot et al. 2010). In higher eukaryotes, Pol III transcription is regulated by several activators and repressors, among them MYC, RB1 and other pocket proteins, TRP53, PTEN, and MAF1 (White 2004, 2008; Bhargava et al. 2013). MAF1, a regulated phosphoprotein conserved from yeast to human (Pluta et al. 2001), represses Pol III transcription in response to diverse cellular and environmental stresses and nutrient deprivation (Upadhya et al. 2002; Reina et al. 2006; Johnson et al. 2007; Cieśla and Boguta 2008; Michels et al. 2010). In mammalian cells, MAF1 is directly phosphorylated by the mammalian TOR kinase complex 1 (mTORC1), and repressing conditions such as serum deprivation and alkylating DNA damage lead to MAF1 dephosphorylation and increase its localization to actively transcribed Pol III genes. Subsequently, Pol III occupancy at many Pol III loci is reduced (Shor et al. 2010; Michels 2011; Kantidakis et al. 2012; Orioli et al. 2016). TORC1 is part of the insulin signaling pathway and constitutes a sensor of nutrient availability; MAF1 may thus serve to adapt Pol III transcription to nutrient availability. Whether Pol III transcription is subjected to diurnal regulation, and the role that MAF1 might play in such regulation, has yet to be determined.

Here, we explored whether Pol III occupancy of its target genes, which reflects transcription activity (Orioli et al. 2016), varies during the diurnal cycle, and we examined the contributions of the circadian cycle and the nutrient response as well as the role of MAF1 in this regulation.

## Results

**Figure 1.**
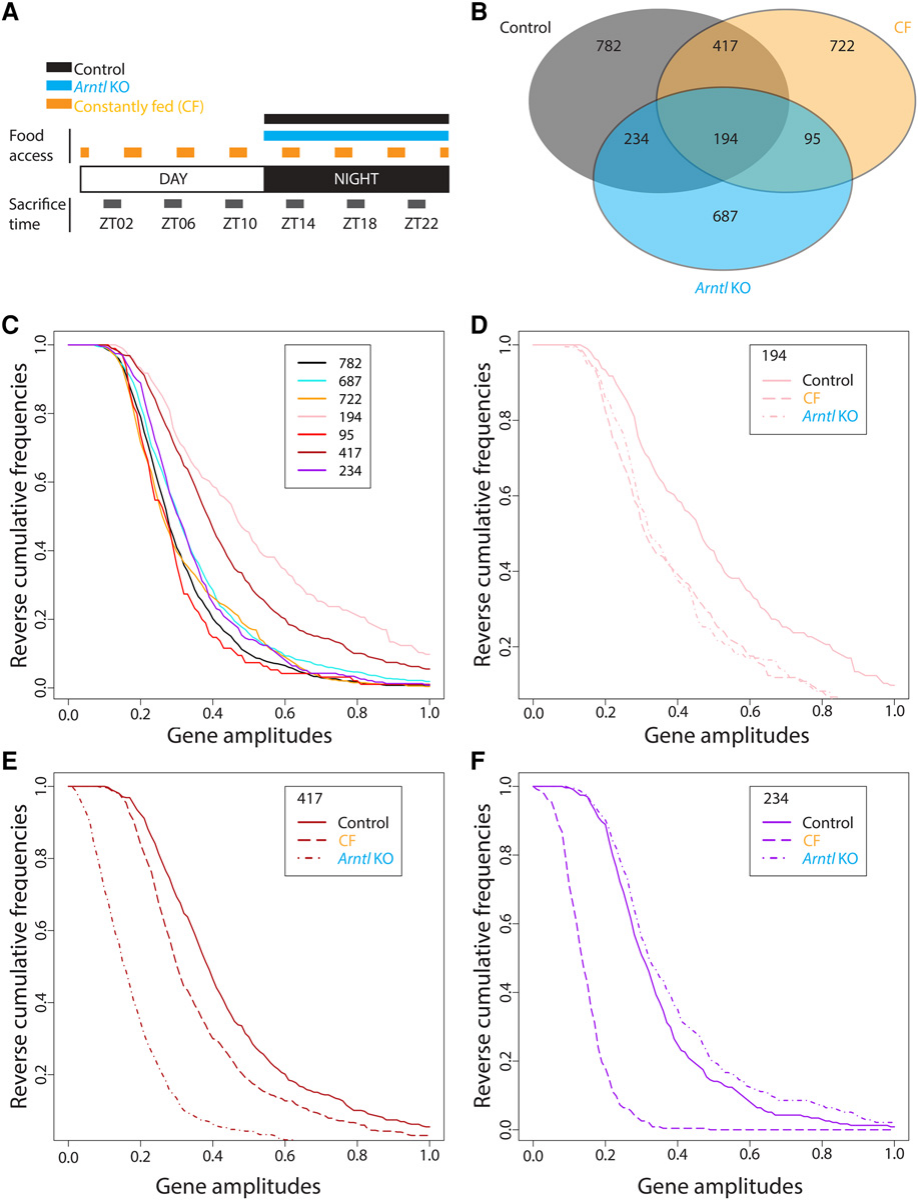
Rhythmic mRNAs in control, CF, and *Arntl* KO mouse liver. (*A*) Experimental design. Mice were fed over the course of 1 wk either only during the night (control and *Arntl* KO mice) or every 3 h (CF, constantly fed mice). They were then sacrificed every 4 h during two consecutive days (3–6 replicates per time point). For CF mice, the timing was established such that sacrifice always occurred 1 h after feeding. Liver RNA was extracted and used for gene expression microarray analyses. A cosine function was fitted to the data, and genes with a *P*-value associated with the fitting to the model lower than 0.0001 were considered as oscillating genes. (*B*) Venn diagram displaying the number of genes oscillating in each condition. The false discovery rates were 0.09 for WT mice, 0.08 for CF mice, and 0.09 for the *Arntl* KO mice (1000 permutations). (*C*) Reverse cumulative frequencies (RCF) of the amplitudes of the indicated group of genes (corresponding to the Venn diagram) in control mice (782, 417, 234, 194), CF (722, 95), and *Arntl* KO (687). RCF corresponds here to the frequency of all the values greater than a given amplitude. (*D*) RCF of the amplitude in the control, CF, and *Arntl* KO mice for the 194 genes oscillating in all the conditions. (*E*) As in *D*, but for the 417 genes oscillating in both control and CF mice. (*F*) As in *D*, but for the 234 genes oscillating in both the control and *Arntl* KO livers.

To assess the contributions of the diurnal cycle and the feeding–fasting cycle to the regulation of Pol III transcription, we sought to disentangle the effects resulting from each of these cycles. We compared liver samples collected every 4 h from wild-type mice (WT, control), mice arrhythmic lacking the *Arntl* gene (*Arntl* KO) (Bunger et al. 2000; McDearmon et al. 2006), and wild-type mice fed at frequent and regular intervals during the entire diurnal cycle (hereafter referred to as “constantly fed” [CF]). All mice were kept under a 12-h light/12-h dark cycle, and both control and *Arntl* KO mice were fed only during the night to avoid any confounding effects resulting from altered feeding behavior in the *Arntl* KO (Fig. 1A). CF mice were housed in special cages that allowed adjustment of food availability; chow powder was sequestered in a compartment closed by a mechanical door, which could be programmed to open at any frequency and for any length of time (Supplemental Fig. S1A). Preliminary experiments led to the regimen described in Methods, in which after a training period, mice consumed food at constant intervals during both day and night without losing weight over the course of the experiment (Supplemental Fig. S1B,C).

To characterize our experimental conditions, we performed gene expression microarray analyses with liver samples from at least three mice for each condition and time point. To identify genes displaying circadian mRNA abundance oscillations, we fitted a cosine function to estimate amplitude and phase and selected genes with cosine fitted *P*-values of ≤0.0001 (Methods; Supplemental Table S1). In the control mice, this method identified 1627 mRNAs as displaying circadian rhythmicity, which we defined as our control rhythmic data set. In the CF and *Arntl* KO samples, the same method identified 1428 and 1210 rhythmic mRNAs, respectively. These sets of rhythmic mRNAs were, however, quite different, with only 194 mRNAs displaying rhythmic oscillations under the three conditions (Fig. 1B). These 194 genes corresponded to those with, on average, the highest amplitudes in the control mice (Fig. 1C), and their amplitudes were strongly diminished in the *Arntl* KO and CF mice (Fig. 1D). On the other hand, mRNAs oscillating only in the control (782), *Arntl* KO (687), or CF (722) mice displayed much lower, and quite similar, amplitudes (Fig. 1C). The 417 genes oscillating only in the control and CF mice, which included all the core clock genes (Supplemental Table S2) had the second highest amplitudes in the control set (Fig. 1C), and these amplitudes were diminished in the CF mice (Fig. 1E). In contrast, the 234 genes oscillating only in the control and *Arntl* KO mice had rather low amplitudes in the control set (Fig. 1C), and these amplitudes were slightly higher in the *Arntl* KO liver compared with control (Fig. 1F). Thus, a set of mRNAs oscillating with high amplitudes in the control continues to oscillate, albeit with lower amplitudes, even in the absence of ARNTL or under conditions of constant feeding: several of these mRNAs code for factors involved in organic acid catabolism and lipid metabolism (Supplemental Table S2). Moreover, some mRNAs oscillate only under CF or *Arntl* KO conditions, consistent with the possibility that restricted feeding and circadian clock cannot only generate, but also dampen, rhythms.

**Figure 2.**
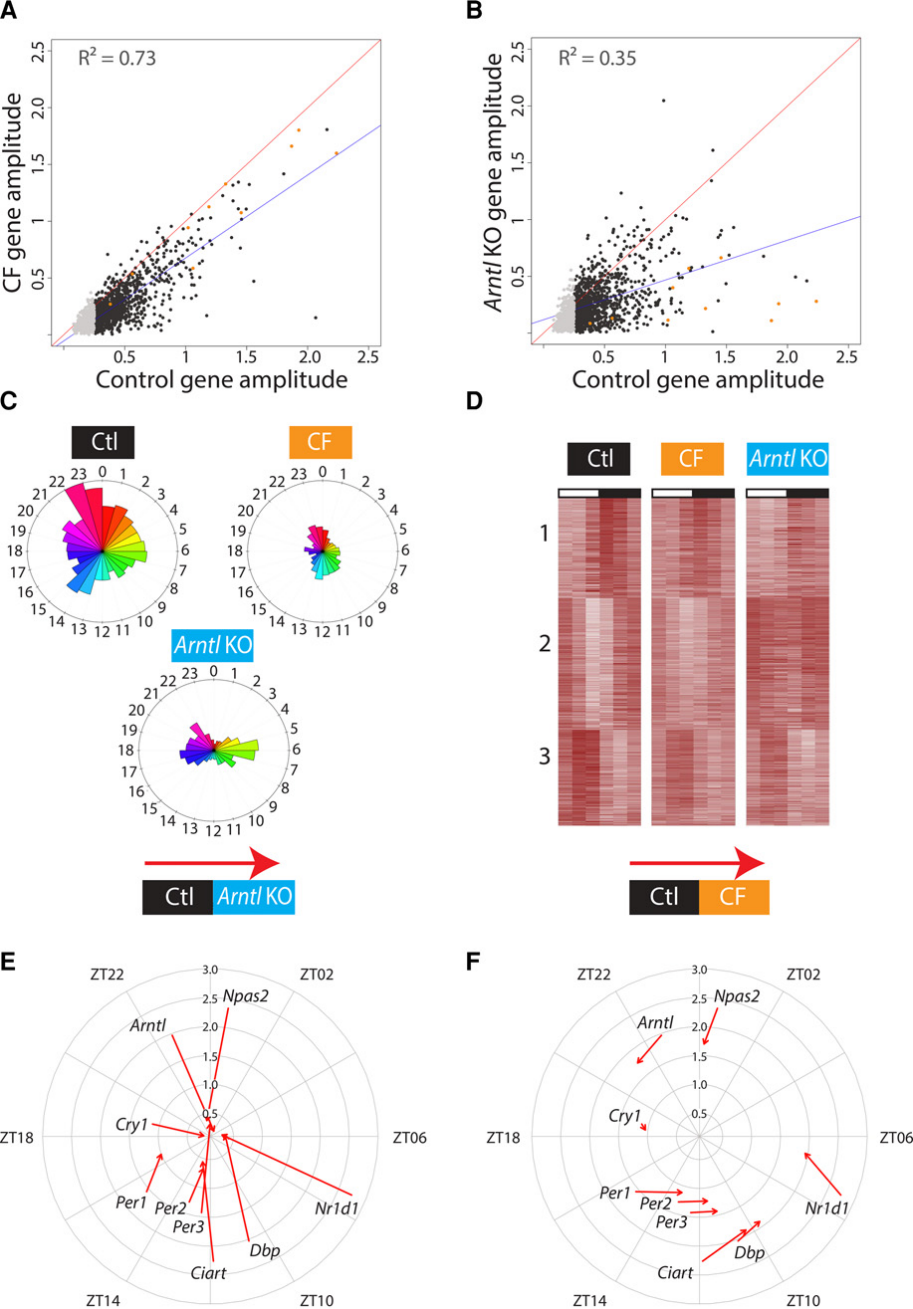
Effects of CF and *Arntl* KO conditions on the genes oscillating in control liver (control rhythmic data set). (*A*) Scatterplot showing amplitudes [(maximum − minimum gene expression)/2] in control (*x*-axis) and CF (*y*-axis) liver, for all genes in the control rhythmic data set with an amplitude higher than 0.25 in the control condition (1132 of 1627 genes, black and orange dots). The light gray dots represent genes with amplitude lower than 0.25 in control condition (495 of 1627 genes). The red line is the X = Y line, and the blue line is the best fit. The orange dots are circadian-related genes, selected according to gene ontology analysis. (*B*) As in *A* but in control (*x*-axis) and *Arntl* KO (*y*-axis) liver. (*C*) The cutoff on the amplitude was applied for each condition on the control rhythmic data set genes resulting in 1132 of 1627 oscillating genes in control mice, 504 of this same set in CF mice, and 582 of this same set in *Arntl* KO mice. For each time of the day (indicated as Zeitgebers), the number of genes from the control rhythmic data set with a corresponding phase (time of maximum gene expression) is represented in control, CF, and *Arntl* KO mice. The radius is equal to the highest number of genes among all three conditions, i.e., 86 genes with a phase between ZT22-ZT23 in the control mice. (*D*) PAM analysis was performed on the 1132 genes from the control rhythmic data set, and the number of clusters was determined by the best silhouette average. Genes were ranked according to the control condition and normalized by row across all conditions: (white) lowest expression; (red) highest expression. (*E*) Circular plot showing the phases around the periphery and the amplitudes along the radius. The arrows show the phase and the amplitude of circadian clock gene expression in control (beginning of arrows) and *Arntl* KO (end of arrows) liver. (*F*) As in *E*, but for circadian clock genes in control (beginning of arrows) and CF (end of arrows) liver.

We then considered the 1627 mRNAs that constitute the control rhythmic data set and applied, in addition to the *P*-value filter, a filter eliminating genes with amplitudes lower than 0.25 (in log_2_, fold change lower than 1.4), which gave us a set of 1132 genes. The amplitudes and the phases of these genes were much better correlated in control and CF conditions than in control and *Arntl* KO conditions (Fig. 2A,B; Supplemental Figs. S2A,B). In the control condition, two main groups of genes had phases at the beginning and at the end of the night (ZT14-15 and ZT22-23) (Fig. 2C); in the CF and *Arntl* KO conditions, only 504 and 582 of these 1132 genes, respectively, still had amplitudes above 0.25; as expected from the phase correlations above, the distribution of their phases was quite similar in the CF condition, but very different in the *Arntl* KO conditions, with prominent groups of genes displaying phases in the middle of the day and the middle of the night (Fig. 2C).

We subjected the 1132 genes of the control rhythmic data set to a PAM (partitioning around medoïds) analysis, selecting the number of clusters according to the best average of silhouette widths (Supplemental Fig. S3A). These genes could be best separated into three clusters, representing genes with peaks of expression at three main time points during the day (Fig. 2D; Supplemental Table S3). Cluster 1 included 347 genes with peaks of expression mostly just after the onset of the night, when food became available. Many of these genes were associated with circadian regulation of gene expression, circadian rhythm, and rhythmic processes (Supplemental Fig. S3B). Cluster 2 was composed of 454 genes with peaks of expression at dawn, the end of the feeding period (ZT23). These genes were mostly associated with lipid metabolic processes (Supplemental Fig. S3C). Cluster 3 included 331 genes that peaked at the end of the day (ZT10) and were associated with steroid metabolic processes, as previously reported (Edwards et al. 1972; Matsumoto et al. 2010; Gilardi et al. 2014), as well as glycogen metabolic process, circadian regulation of gene expression, circadian rhythm, and rhythmic processes. The rhythmic expression of these genes was altered in both CF mice and *Arntl* KO mice, although to a greater extent in the latter, as illustrated by blurring of the clusters (Fig. 2D, middle and rightmost panels), consistent with the results above. Thus, genes involved in metabolic processes displayed widespread rhythmic expression changes not only in the CF mice but also in the *Arntl* KO mice, consistent with many of the liver CCGs corresponding to genes involved in metabolism (Mazzoccoli et al. 2012; Rey and Reddy 2013). Moreover, the amplitudes and phases of the 1132 genes oscillating under control conditions were globally more affected in the *Arntl* KO mice than in the CF mice. Of note, 171 mRNAs from the rhythmic control data set showed higher amplitude in the *Arntl* KO mice, of which several had lipid and cholesterol regulation, as well as insulin response, as associated GO terms (Supplemental Table S4). This suggests that one of the functions of ARNTL is to dampen rhythms related to the nutrient response.

### *Arntl* KO mice, but not CF mice, lose rhythmic expression of core clock genes

To examine specifically how the core circadian clock was affected in the various conditions, we examined core clock and other well-studied circadian genes individually. The *Per1-3*, *Cry1*, *Arntl*, *Npas2*, *Nr1d1*, *Dbp*, and *Ciart* genes displayed the expected loss of rhythmicity in the *Arntl* KO mice as shown by greatly reduced amplitudes of mRNA accumulation, but had only slightly reduced amplitudes and a slightly advanced phase (1.5 h on average) in CF mice (see Fig. 2A,B, orange dots; Fig. 2E,F). Thus, the rhythmic expression of core circadian clock genes is strongly affected in *Arntl* KO mice, as expected, but left mostly undisturbed in CF mice.

### Constantly fed mice lose night-specific blood insulin elevation and TORC1 pathway activation

**Figure 3.**
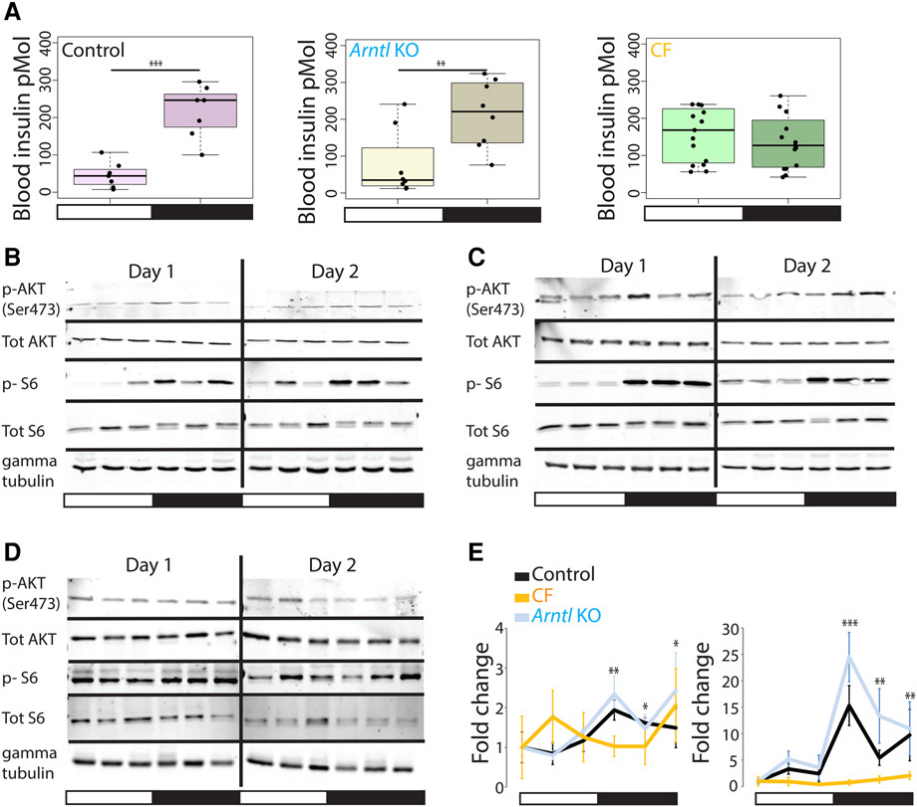
Effects of CF and *Arntl* KO conditions on nutrient-response mediators. (*A*) Night and day blood insulin concentration in control, *Arntl* KO, and CF mice. Each box plot represents the distribution of blood insulin concentration at different time points during the day (ZT02, ZT06, ZT10) and the night (ZT14, ZT18, ZT22). The horizontal black line is the median, and each point is the value for a given mouse: (***) *P*-value <0.0005; (**) *P*-value <0.005. (*B*) Immunoblots of control mouse liver samples collected every 4 h for 2 d and probed with antibodies directed against the antigens indicated on the *left*: p-AKT, AKT phosphorylated on serine 473, tot AKT, total AKT; p-S6, S6 phosphorylated on serines 235/236; Tot S6, total S6. Gamma tubulin was used as loading control. (*C*) As in *B*, but with *Arntl* KO mice liver samples. (*D*) As in *B*, but with CF mice liver samples. (*E*) Quantitation of the immunoblot signal fold changes obtained for phosphorylated AKT/Total AKT (*left*) and phosphorylated S6/Total S6 (*right*). Mean immunoblot signals were calculated for control (black, 3–5 samples per time point), *Arntl* KO (blue, 3–5 samples per time point), and CF (orange, 4–6 samples per time point) mice, and the signal at ZT02 was set at 1. For each condition, *P*-values were calculated for each night time point compared with the mean of all the day time points: (***) *P*-value <0.0005; (**) *P*-value <0.005; (*) *P*-value <0.05.

The gene expression array data above suggested broad deregulation of genes involved in various metabolic processes in both the *Arntl* KO and CF mice (Fig. 2D; Supplemental Table S3). Food ingestion leads to the secretion of insulin by the β-cells of the pancreas and increased blood insulin concentration. Binding of insulin to its tyrosine kinase receptor then generates a cascade of phosphorylation events involving intermediate kinases such as AKT and TORC1, which play major roles in mediating insulin functions (Manning 2004; Laplante and Sabatini 2009). To determine how this insulin response might be altered in our experimental system, we measured blood insulin concentrations by immunoassays at different times during the diurnal cycle, and then compared day and night time points. Control and *Arntl* KO mice exhibited higher blood insulin concentration at night, when they had access to food, whereas CF mice showed no significant difference in blood insulin concentration between day and night (Fig. 3A). We prepared liver homogenates to monitor the phosphorylation state of AKT (serine 473), reflecting AKT activation, and S6 ribosomal protein (serines 235/236), reflecting TORC1 activity. In control mice, both proteins were more highly phosphorylated during the night; for AKT, the day–night difference was slight, with a phosphorylation peak at ZT14, whereas the difference was more pronounced for S6 (Fig. 3B,E). In the *Arntl* KO mice, the day–night differences were amplified relative to control mice, especially for S6, whose phosphorylation was induced very strongly during the night (Fig. 3C,E). In contrast, CF mice showed no systematic changes in AKT and S6 protein phosphorylation between day and night (Fig. 3D,E). Thus, blood insulin levels and insulin pathway activity were consistent with control and *Arntl* KO mice eating at night, when food was available, and CF mice eating during both night and day. Further, the increased insulin pathway activity of the *Arntl* KO mice relative to control was consistent with previous observations that *Arntl* KO mice are hypersensitive to insulin and display increased TORC1 activity (Lamia et al. 2008).

Altogether, these data show that in CF mice, the diurnal regulation of the AKT-TORC1 nutrient response, but not that of the core circadian clock, gene expression is lost; in *Arntl* KO mice, the diurnal regulation of the AKT-TORC1 nutrient response is, if anything, increased, whereas circadian clock gene expression is lost. The experimental system thus allows us to affect separately the AKT-TORC1 nutrient response and the core circadian clock.

### Increased overall Pol III gene occupancy in CF and *Arntl* KO mouse liver

**Figure 4.**
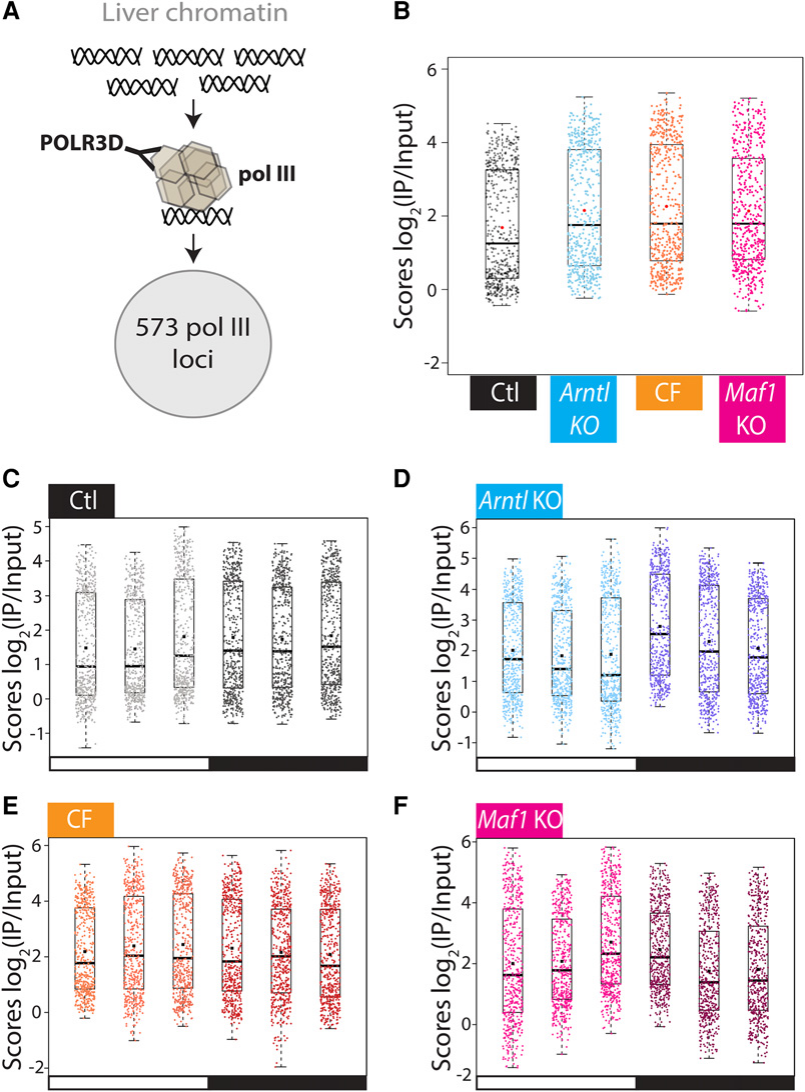
Pol III gene occupancy increase in CF, *Arntl* KO, and *Maf1* KO mice. (*A*) ChIP-seq was performed with an antibody directed against POLR3D (RPC4), a Pol III subunit, with pools of three liver samples from 12- to 14-wk-old mice collected every 4 h during two consecutive days. Five hundred seventy-three loci significantly occupied by Pol III in at least one sample in one of the conditions were analyzed. (*B*) Box plot showing, for each of the 573 loci, the mean of all scores [log_2_(IP/Input)] in all time points (12 samples per condition) for control, CF, *Arntl* KO, and *Maf1* KO liver samples. The mean and median of the 573 loci are represented with a black squared dot and a black line, respectively. (*C*) Box plot showing the mean score of two biological replicates at each time point for the 573 loci. (*D*) As in *C*, but for the *Arntl* KO mice. (*E*) As in *C*, but for the CF mice. (*F*) As in *C*, but for the *Maf1* KO mice.

To assess the effect of the circadian clock and the feeding–fasting response on Pol III recruitment throughout the day, we determined Pol III occupancy in the experimental system described above by ChIP-seq using an antibody against the Pol III subunit POLR3D (also known as RPC4) (Fig. 4A; Supplemental Table S5; Canella et al. 2010). The samples were spiked with a small, constant amount of human chromatin for sample-to-sample normalization (Bonhoure et al. 2014). We analyzed a set of loci either annotated as Pol III genes in the genome assembly or found significantly occupied by Pol III in one of our previous ChIP-seq analyses (Canella 2012; Bonhoure et al. 2014; Renaud 2014). Among these loci, we selected those significantly occupied by Pol III in at least one sample in one of the conditions. We first examined the mean score for all samples collected for each condition (two replicates for each of the six time points, i.e., 12 samples/condition). The mean Pol III occupancy was significantly higher in *Arntl* KO and CF mice compared with control mice (Fig. 4B), suggesting that one of the functions of ARNTL is to repress, directly or indirectly, overall Pol III occupancy, and that constant food ingestion increases overall Pol III occupancy.

### Increased Pol III occupancy during the night in control and *Arntl* KO, but not CF mice

**Figure 5.**
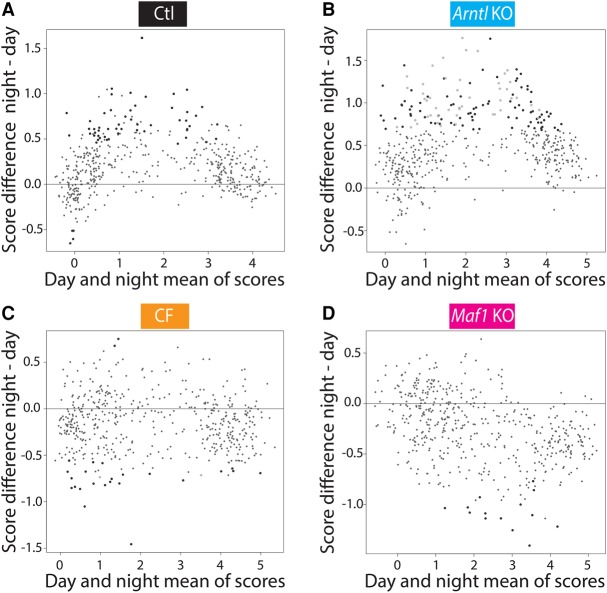
Nutrient-dependent increase of Pol III gene occupancy. (*A*) MVA plot comparing day and night average scores. For each locus, the three day scores were averaged and the three night scores were averaged. The MVA plot shows the average of day averages and night averages on the *x*-axis and the difference of night average – day average scores on the *y*-axis. Large black dots represent genes with significantly different day average and night average scores (*P*-value <0.05), as calculated with limma. (*B*) As in *A*, but for *Arntl* KO mice. The large black dots represent genes changing with a *P*-value <0.05 only in the *Arntl* KO mice, and the large gray dots represent genes changing with a *P*-value <0.05 in both the control and the *Arntl* KO mice. (*C*) As in *A*, but for the CF mice. The large black dots represent genes changing with a *P*-value <0.05 only in the CF mice, and the large gray dots represent genes changing with a *P*-value <0.05 in both the control and CF mice. (*D*) As in *A*, but for the *Maf1* KO mice. The large black dots represent genes changing with a *P*-value <0.05 only in the *Maf1* KO.

We then examined Pol III occupancy at the different time points and in the different conditions. We first tested whether the occupancy scores could be fitted to a cosine curve and observed that this was the case for only 6%–16 % of Pol III loci at a *P*-value lower than 0.05. On the other hand, in both the control and *Arntl* KO mice, the mean Pol III occupancy scores appeared globally higher during the night than during the day (although there were differences at ZT10, the last time point of the day, see below), whereas CF mice showed little difference between day and night (Fig. 4C–E). To examine more closely this day–night difference, we compared intra-group variance of daytime samples (considered here as replicates) and nighttime samples (considered here as replicates) to the intergroup variance of day and night samples with limma (Ritchie et al. 2015). This confirmed a tendency for most of the Pol III loci in the control mice toward increased Pol III occupancy during the night, with 58 loci more occupied considering a *P*-value <0.05 (large black dots in Fig. 5A). In the *Arntl* KO mice, the difference was larger compared with the control mice, with 133 genes displaying significantly higher Pol III occupancy during the night. This gene set included most (46) of the 58 genes displaying higher nighttime occupancy in the control mice (Fig. 5B). In contrast, no increase of Pol III occupancy was observed during the night in the CF mice; in fact, of 27 genes whose occupancy was significantly affected, most showed a decrease during the night (Fig. 5C). Thus, both control and *Arntl* KO mice showed increased Pol III occupancy during the night, when food was available, whereas CF mice, which consumed food during both night and day, did not show this increase. This is consistent with the high nighttime Pol III occupancy occurring in response to food intake.

### Anticipation of night period in the liver of control and CF, but not of *Arntl* KO, mice

**Figure 6.**
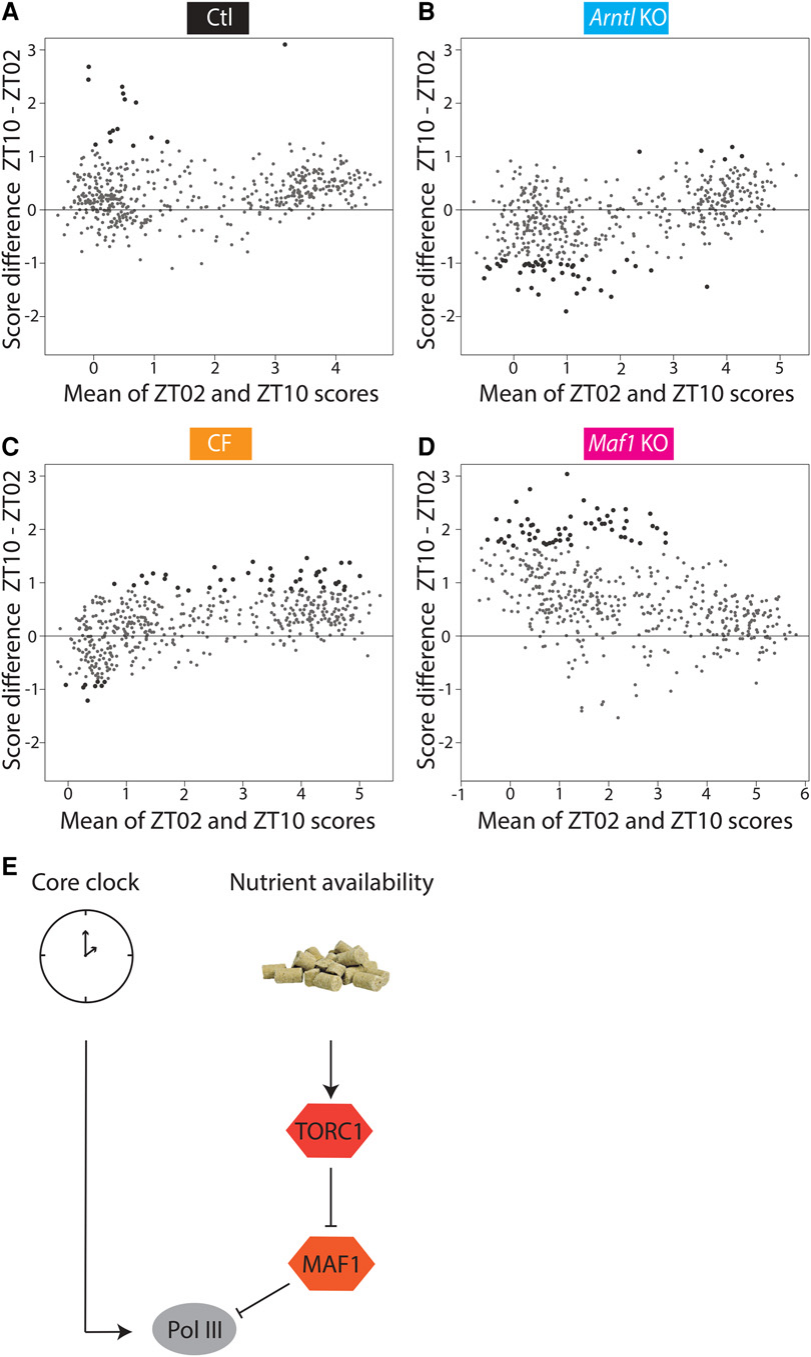
The circadian clock enables nutrient-independent Pol III recruitment. (*A*) MVA plot showing the average of ZT02 and ZT10 scores on the *x*-axis and the difference of ZT10 – ZT02 scores on the *y*-axis. Large black dots represent genes changing between day and night with a *P*-value <0.05, as calculated with limma. (*B*) As in *A*, but for *Arntl* KO samples. (*C*) As in *A*, but for the CF samples. (*D*) As in *A*, but for the *Maf1* KO samples. (*E*) Regulation of Pol III gene occupancy by the core clock, which activates Pol III before the night feeding period, and by the feeding response, which activates TORC1, which itself inactivates the MAF1 repressor.

When we considered individual time points, specifically when we compared the beginning and the end of the light period (ZT02 versus ZT10), we confirmed the general tendency visible in Figure 4C,E toward increased Pol III occupancy in the control (17 genes with a *P*-value <0.05) as well as in the CF (41 genes with *P*-value <0.05) mice at the last time point of the day (Fig. 6A,C). In contrast, in the *Arntl* KO mice, there was no global increase. In fact, more loci showed decreased rather than increased Pol III occupancy (45 genes down and five genes up with *P*-value <0.05) (Fig. 6B). Thus in control mice and CF mice, which have a functional circadian clock, Pol III occupancy started to rise before the dark period, whereas in the *Arntl* KO, it started to rise after the beginning of the dark period and availability of food. This suggests that the core circadian clock contributes to the temporal regulation of Pol III occupancy leading to increased Pol III occupancy in preparation for the dark period, when control mice are normally active and ingest food.

### MAF1 contributes to regulation of Pol III occupancy by the nutrient response

The preceding results suggest that temporal Pol III occupancy is determined by two mechanisms: a core clock-dependent mechanism allowing increased Pol III occupancy just before the night (i.e., except for the CF mice, the feeding period) and a nutrient-response mechanism allowing increased Pol III occupancy during the feeding period. To determine whether the Pol III repressor MAF1 plays a role in these regulation mechanisms, we collected liver samples from *Maf1* KO mice fed only during the night. We first examined the behavior of the Pol II genes in the control rhythmic data set. Compared with the *Arntl* KO and CF conditions, the amplitudes and phases in the *Maf1* KO samples were the most highly correlated with those in the control condition (Supplemental Fig. S4A,B). The phases of mRNA accumulation were only slightly changed; specifically many genes with a phase between ZT22 and ZT24 in the control had a phase between ZT00 and ZT02 in the *Maf1* KO (Supplemental Fig. S4B,C; Supplemental Table S1). Indeed, PAM analysis revealed a pattern very similar to that obtained in the control conditions (Supplemental Fig. S4D), and specific examination of the core circadian clock genes revealed slightly delayed phases and little changes in amplitude (Supplemental Fig. S4E).

Contrasting with the relatively small effects on the control rhythmic data set, the absence of MAF1 resulted in an overall increase in Pol III gene occupancy compared with control mice, similar to that observed in CF mice and *Arntl* KO mice (Fig. 4B). This observation is consistent with previous results obtained in *Maf1* KO mouse liver (Bonhoure et al. 2014) and with the recent observation that MAF1 is a chronic repressor of Pol III in human cells (Orioli et al. 2016). Moreover, the Pol III occupancy pattern at the different time points showed an increase at the end of the day and beginning of the night (Fig. 4F), but a comparison of the averaged day and the averaged night time points revealed no increased occupancy during the night; in fact 14 genes showed a significant decrease (Fig. 5D). This suggests that in the *Maf1* KO mice, the feeding–fasting regulation, which results in lower Pol III occupancy during most of the day, is lost, consistent with this lower daytime occupancy being normally caused by the known inactivation of the MAF1 repressor by the TORC1 pathway. We then compared Pol III gene occupancy at the beginning and end of the day and observed a clear increase at the end of the day, with 58 genes showing a significant increase (Fig. 6D). This is consistent with the idea that the core clock-dependent regulation, which causes Pol III occupancy to rise in preparation for the night (and feeding) period, is maintained. Thus, the feeding–fasting regulation is largely MAF1-dependent, whereas the end of day increase before the feeding period is largely MAF1-independent.

## Discussion

Pol III transcription has been shown to be tightly linked to cell growth and proliferation, both in yeast and in mammals (White 2004, 2008; Vannini et al. 2010; Bhargava et al. 2013; Moir and Willis 2013, 2015), and is repressed following nutrient limitation, DNA damage, rapamycin treatment, and other stresses in a process that requires the MAF1 protein (Upadhya et al. 2002; Reina et al. 2006; Michels et al. 2010). Here, we have explored whether Pol III transcription is regulated during the diurnal cycle in mouse liver, whether any such regulation might reflect a response to nutrients, and whether it might be dependent on the MAF1 protein. To do so, we first characterized the experimental system by examining differences in rhythmic mRNA accumulation as measured by microarray assays under the different conditions.

### Rhythmic genes in WT, CF, and *Arntl* KO conditions

Microarray analysis of liver RNA from control mice identified 1627 rhythmic genes with two main phases, at the beginning and the end of the night. This is consistent with a number of previous studies that have defined variously sized sets of rhythmic genes, often with two main phases, although the phase values vary from study to study. This variation probably results from different analysis pipelines, but also from different experimental conditions: for example, constant darkness or constant dim light versus a 12-h day/12-h night regimen, or ad libitum feeding versus night-restricted feeding (Panda et al. 2002; Storch et al. 2002; Miller et al. 2007; Le Martelot et al. 2012; Menet et al. 2012; Zhang et al. 2014; Atger et al. 2015). Indeed, restricted feeding in particular is known to increase the number as well as the amplitudes of cycling transcripts (Vollmers et al. 2009; Hatori et al. 2012; Atger et al. 2015). Among the 1627 genes, a set of 194, several of them involved in catabolic processes, oscillated in all conditions, albeit with much higher amplitudes in WT mice than in CF or *Arntl* KO conditions. These genes are probably regulated by a number of concordant rhythmic signals, at least one of which remains active in the *Arntl* KO mice and the CF mice.

The sets of genes oscillating in the CF and *Arntl* KO samples were quite different from the set in WT samples. In particular they contained newly oscillating genes, albeit with relatively low amplitudes, associated with stress-related processes such as positive regulation of TRP53 in the CF case, and basal mechanisms such as DNA replication and ribosome biogenesis in the second case (Supplemental Table S2). These genes may normally be subjected to at least two rhythmic signals, one linked to night-restricted feeding and the other to a functional core circadian clock, that counteract each other and are thus revealed only when one of the two signals is abolished. The phases of these signals may be different, or one signal may be activating and the other repressing.

### The nutrient response cycle and the circadian clock in CF and *Arntl* KO samples

Expression of the core clock genes was, as expected, greatly affected in *Arntl* KO mice, with large losses of amplitudes, but largely unaffected in CF mice, where the main effect was a slight (1.5 h) phase advance. This is consistent with the previous observation of core clock gene phase advances in mice subjected to ultradian feeding cycles (feeding cycles with a period shorter than 24 h), although the previously observed phase shifts were larger (4–6 h), perhaps because unlike in our case, the mice were calorie-restricted (van der Veen et al. 2005; Sen et al. 2017).

The nutrient response involves notable changes in plasma insulin levels and activation of the AKT and TORC1 pathways. TORC1 activation in turn leads to activation of translation, notably translation of TOP mRNAs, which encode components of the translation apparatus and ribosomes (Thoreen et al. 2012). We found that day–night changes in both plasma insulin levels and in TORC1 activation were maintained in the *Arntl* KO mice under night-restricted feeding conditions; in fact, TORC1 activation during the night was, if anything, stronger in *Arntl* KO mice than in control mice. Moreover, some of the pathways represented among the genes oscillating only in the *Arntl* KO mice were linked to translation and ribosome biogenesis (Supplemental Table S2). The rhythmic activation of TORC1 in *Arntl* KO mouse liver may seem contradictory to previous results, which have shown liver TORC1 activation to be under circadian clock control; rhythmicity was maintained under constant darkness and under conditions of starvation (Jouffe et al. 2013), and translation and ribosome biogenesis was dependent on the circadian clock in various organisms (Dong et al. 2008; Piques et al. 2009; Xu et al. 2011; Jouffe et al. 2013; Khapre et al. 2014). More recent results, however, have pointed to a pivotal role of feeding rhythms in rhythmic translation of TOP mRNAs (Atger et al. 2015). Our results clearly show that the TORC1 pathway in the liver can be activated by feeding in the absence of a functional core clock; furthermore, ultradian feeding in mice with an intact circadian clock leads to more or less constant TORC1 activation with no change between day and night, correlating with a flattened blood insulin profile. This suggests that the response to feeding is largely dominant over any circadian control for liver TORC1 activation.

### Control of Pol III gene occupancy by both the circadian clock and the feeding response

In control mice fed only during the night, Pol III occupancy, as summarized by the mean of the ChIP-seq scores, rose during the day to reach a maximum at the end of the day, after the peak of ARNTL binding to its targets (Koike et al. 2012), and remained high during the night. A comparison with the dynamics of Pol III occupancy in different genotypes and conditions suggests that this pattern reflects two combined modes of regulation—one corresponding to a response to food ingestion and the other corresponding to a circadian regulation independent of food ingestion, as illustrated in Figure 6E. Thus, in mice that lacked ARNTL and, therefore, a functional clock, Pol III occupancy was strongly increased at the beginning of the night rather than at the end of the day, consistent with Pol III recruitment in response to, but not in anticipation of, food ingestion. Moreover, *Arntl* KO mice displayed an overall increase in Pol III occupancy and accentuated day–night differences, suggesting that a functional clock serves not only to prepare the liver for food ingestion but also to keep Pol III transcription in check and to dampen its variation.

In constantly fed mice, there was no overall difference in average Pol III occupancy during day and night; nevertheless the increased occupancy at the end of the day, before the feeding period, was present. These mice then seem to retain a mechanism, lost in the *Arntl* KO mice, that leads to higher occupancy in anticipation of the feeding period. This mechanism, which does not correspond to a nutrient response and is dependent on ARNTL, is most likely a circadian-clock directed response.

### Pol III regulation in *Maf1* KO mice

In human cultured cells, MAF1 can be found on Pol III genes not only after serum starvation but also, although to a lesser extent, under serum-replete conditions, suggesting that MAF1 keeps Pol III transcription in check even under optimal growth conditions (Orioli et al. 2016). Here, we observed that overall Pol III gene occupancy was increased in the *Maf1* KO mice, both during night and day, i.e., during both fasting and feeding period, confirming the idea that MAF1 serves to limit Pol III transcription under a wide variety of conditions. Nevertheless, MAF1 is clearly a major mediator of Pol III transcription regulation in response to nutrients, controlled by the insulin-TORC1 signal transduction pathway. Thus, in yeast, Maf1 binding to Pol III loci correlates with Pol III transcription repression in the absence of nutrients or exposure to rapamycin (Oficjalska-Pham et al. 2006; Roberts et al. 2006). And in mammalian cells, Pol III occupancy is decreased after serum withdrawal, an effect strongly attenuated upon siRNA-mediated knockdown of *Maf1*, and Pol III occupancy increases again upon addition of insulin (but not upon addition of insulin together with rapamycin) (Orioli et al. 2016). In the experimental system used here, we observe higher Pol III occupancy during the night in both control and *Arntl* KO mice, but not in CF mice, strongly suggesting that in the animal, major regulation of Pol III is in response to food intake. Consistent with this picture, *Maf1* KO mice, like the CF mice, did not display increased Pol III occupancy during the night although they had access to food only during the night. Instead, Pol III occupancy was high even during the day. Nevertheless, there was a clear increase at the end of the day, consistent with ARNTL-dependent higher Pol III occupancy in anticipation of the feeding period still being functional. Our results here show that MAF1 is partly inactivated in response to feeding and thus corresponds to a mediator of the feeding–fasting response in the animal (Fig. 6E).

### Concerted regulation of components of the translation apparatus

Translation efficiency of certain mRNAs, as determined by ribosome profiling, is regulated during the diurnal cycle (Atger et al. 2015). A striking example is that of the TOP mRNAs, which encode the protein components of the ribosome, and which are most highly translated during the night (Jouffe et al. 2013; Atger et al. 2015). Moreover, transcription of 45S RNA, the precursor of the large ribosomal RNA, is also activated during the night (Jouffe et al. 2013). Our results show that yet other components of the translation apparatus, synthesized by Pol III, are rhythmically synthesized (as measured by Pol III occupancy) (Orioli et al. 2016) with peak expression during the night, and that this effect results in part because MAF1-mediated repression of Pol III transcription is decreased during that time. As MAF1 is directly phosphorylated and thus inactivated by TORC1 (Wei et al. 2009; Kantidakis et al. 2010; Michels et al. 2010), these combined observations show that TORC1 activation during the night coordinates the rhythmic synthesis of the entire translation apparatus.

## Methods

### Animals

Control and *Arntl* KO (Jouffe et al. 2013) mice were housed under a 12-h light/12-h dark regimen with food and water ad libitum for 3 wk. Then, the food was accessible only during the nighttime, from 7 p.m. to 7 a.m., for at least 1 wk before sacrifice (see Fig. 1A). Constantly fed mice were housed under a 12-h light/12-h dark regimen with food and water ad libitum for 2 wk in special cages for acclimatization (Supplemental Fig. S1A). The cages contained a compartment for storage of chow powder closed by a door wired to a computer-controlled motor, which could be programmed to set opening and closing times (Supplemental Fig. S1). Preliminary week-long tests established that allowing access to food for 11 min every 3 h led to weight loss but trained the mice, which were hungry, to feed every time the door opened (with a noise); this regimen could be followed by a regimen allowing feeding for 16 min every 3 h, during which mice continued to feed at every door opening but displayed normal weight gain. The food was thus made available during 11 min every 3 h for a 2-d training period followed by at least 5 d during which food was made available during 16 min every 3 h.

### Liver extracts and Western blotting

Livers were homogenized in RIPA buffer containing Complete Mini and PhosSTOP (Roche). Samples were centrifuged at 14,000*g* for 15 min at 4°C, the interphase was transferred to a new tube, and protein concentration was determined by the BCA assay (Pierce). For Western blotting, the membranes were probed with anti-AKT and anti-phospho-AKT (antibodies 9272 and 9271S from Bioconcept), anti-S6 ribosomal protein and its form phosphorylated on serines 235/236 (antibodies 2217 and 2211S from Bioconcept), and anti-gamma tubulin (antibody 16504 from Abcam) antibodies.

### Microarrays

Pieces of perfused livers (50–100 mg) were added to 1 mL of TRIzol (Life Technologies), mechanically homogenized, and RNA was extracted according to the manufacturer's instructions. Samples from three mice for the control, the *Arntl* KO, and the *Maf1* KO conditions and from 5–6 mice for the CF condition were processed. RNA concentration was determined with a NanoDropND-1000 spectrophotometer, and RNA quality was assessed with the Fragment Analyzer (Advanced Analytical Technologies). For each sample, 100 ng of total RNA was then amplified with the Ambion WT Expression Kit (4411973, Life Technologies). The resulting amplified cDNA material (5.5 µg) was fragmented and labeled with GeneChip WT Terminal Labeling kit (901525, Affymetrix). Affymetrix Mouse Gene 2.0ST arrays were hybridized with 3.7 µg of the fragmented and labeled cDNA material at 45°C for 16 h, then washed and stained according to the manufacturer's protocol (Affymetrix GeneChip Expression Analysis Manual, Fluidics protocol FS450_0002).

The arrays were scanned with the GeneChip Scanner 3000 7G (Affymetrix). Normalized expression signals were calculated from Affymetrix CEL files by the Robust Multi-array Average algorithm (RMA), with the Affymetrix Expression Console Software (version 1.3.1.187). Hybridization quality was assessed with Expression Console Software.

### Rhythmicity analysis and gene selections

To estimate the phase and amplitude of mRNA accumulation, we fitted a harmonic regression with a 24-h period to the normalized microarray expression signals and to the Pol III ChIP-seq scores (Equation 1). Genes were considered as rhythmic if the *P*-value associated with the *F*-statistic of the model represented by Equation 1 was lower than 0.0001.

(1) $$y=b0+b1\times \cos\left(t\times \frac{2\pi }{24}\right)+b2\times \sin\left(t\times \frac{2\pi }{24}\right)+e.$$

Equation 1 is the regression model that was fitted to the gene expression profiles, in which *b*0 represents the mean signal, *b*1 and *b*2 are the coefficients of the cosine and sine functions. The amplitude and the phase were estimated as (sqrt(*b*1^2^ + *b*2^2^)) and (atan(*b*1/*b*2)*24/(2*π)), respectively. The mean signal is obtained by calculating the mean of the expression levels of every gene throughout the day. The amplitude is the distance between the highest expression level and the lowest expression level divided by 2. The phase is the time of the day when a gene has its maximal expression.

### Chromatin immunoprecipitation and sequencing

Preparation of chromatin immunoprecipitation and libraries, high-throughput sequencing, and Pol III occupancy score calculations are as described in Bonhoure et al. (2014).

## Data access

The data in this study have been submitted to the NCBI Gene Expression Omnibus (GEO; https://www.ncbi.nlm.nih.gov/geo/) under accession number GSE89837.

## CycliX Consortium

Nouria Hernandez,^7^ Mauro Delorenzi,^8^,^9^,^10^ Bart Deplancke,^11^ Béatrice Desvergne,^7^ Nicolas Guex,^12^ Winship Herr,^7^ Felix Naef,^11^ Jacques Rougemont,^13^ Ueli Schibler,^14^ Teemu Andersin,^14^,^15^ Pascal Cousin,^7^ Federica Gilardi,^7^ Fabienne Lammers,^7^ François Mange,^7^ Dominic Villeneuve,^7^ Fabrice David,^8^,^13^ Roberto Fabbretti,^12^ Philippe Jacquet,^8^,^13^ Irina Krier,^11^ Dmitry Kuznetsov,^12^ Marion Leleu,^8^,^13^ Robin Liechti,^12^ Olivier Martin,^12^ Eugenia Migliavacca,^7^,^12^,^16^ Aurélien Naldi,^7^,^17^ Viviane Praz,^7^,^8^ Leonor Rib,^7^ Jonathan Sobel,^11^ Volker Vlegel,^12^ Ioannis Xenarios,^7^,^8^,^12^

## Supplementary Material

Supplemental Material

## Footnotes

7 Center for Integrative Genomics, Faculty of Biology and Medicine, University of Lausanne, 1015 Lausanne, Switzerland

8 Swiss Institute of Bioinformatics, 1015 Lausanne, Switzerland

9 Bioinformatics Core Facility, Swiss Institute of Bioinformatics, 1015 Lausanne, Switzerland

10 Department of Oncology and Ludwig Center for Cancer Research, Faculty of Biology and Medicine, University of Lausanne, 1011 Lausanne, Switzerland

11 Interfaculty Institute of Bioengineering, School of Life Sciences, Ecole polytechnique Fédérale de Lausanne, 1015 Lausanne, Switzerland

12 Vital IT, Swiss Institute of Bioinformatics, 1015 Lausanne, Switzerland

13 Bioinformatics and Biostatistics Core Facility, School of Life Sciences, Ecole polytechnique Fédérale de Lausanne, 1015 Lausanne, Switzerland

14 Department of Molecular Biology, Faculty of Sciences, University of Geneva, 1211 Geneva, Switzerland

Present addresses: ^15^Dualsystems Biotech AG, 8952 Schlieren, Switzerland; ^16^Nestlé Institute of Health Sciences, 1015 Lausanne Switzerland; ^17^University of Montpellier, 34095 Montpellier, France
