# Supplementary material for: Diurnal regulation of RNA polymerase III transcription is under the control of both the feeding–fasting response and the circadian clock
Source: Genome Res. 2017 Jun;27(6):973–84. doi: 10.1101/gr.217521.116 (PMC5453330; doi:10.1101/gr.217521.116)
Supplement: Supplemental Material [file supp_27_6_973__index.html]

Diurnal regulation of RNA polymerase III transcription is under the control of both the feeding–fasting response and the circadian clock — Supplemental Material 

# Diurnal regulation of RNA polymerase III transcription is under the control of both the feeding–fasting response and the circadian clock

## Supplemental Material

- Supplemental\_Fig\_S1.pdf
- Supplemental\_Fig\_S2.pdf
- Supplemental\_Fig\_S3.pdf
- Supplemental\_Fig\_S4.pdf
- Supplemental\_Table\_S1.xls
- Supplemental\_Table\_S2.xls
- Supplemental\_Table\_S3.xls
- Supplemental\_Table\_S4.xls
- Supplemental\_Table\_S5.xls
