# Supplementary material for: Diurnal regulation of RNA polymerase III transcription is under the control of both the feeding–fasting response and the circadian clock
Source: Genome Res. 2017 Jun;27(6):973–84. doi: 10.1101/gr.217521.116 (PMC5453330; doi:10.1101/gr.217521.116)
Supplement: Supplemental Material [file supp_gr.217521.116_Supplemental_Fig_S1.pdf]

A

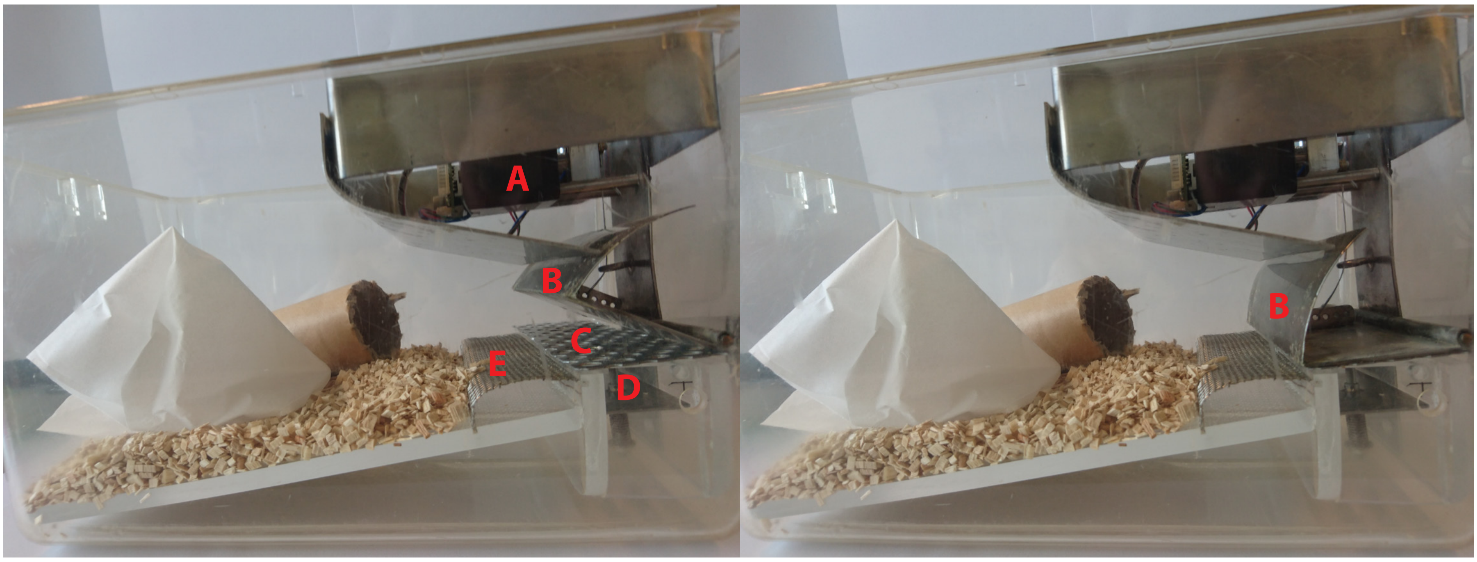

A Motor

B Door (open position)

B Door (closed position)

C Eating area

D Food compartment (spring-loaded platform)

E Grid area

B

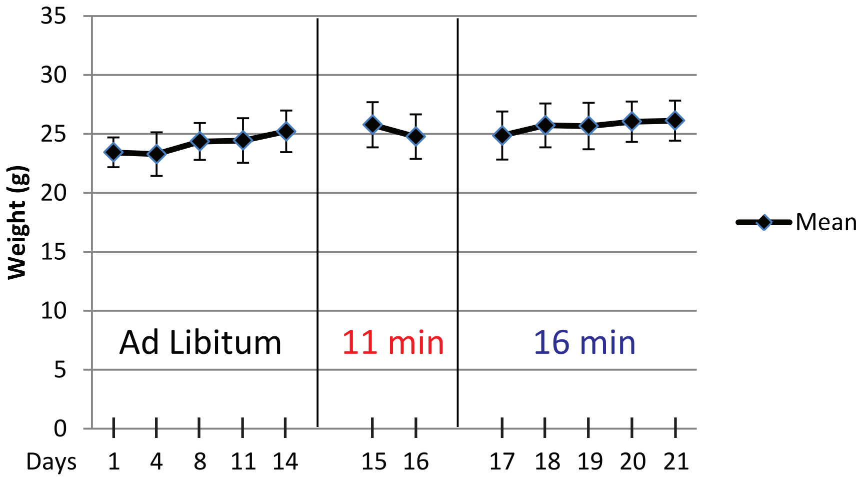

C

Days	4	8	14	16	21
Weight (g)	23.43	24.35	25.21	24.76	26.11
Std	1.27	1.55	1.76	1.88	1.69

Supplemental Fig. S1. Restricted feeding cages.

(A) Side view of a cage used for the CF condition. On the right of the cage, the motorized cover in an open (left photograph) or closed (right photograph) position allowing controlled access to food. The use of powdered food together with the grid in front of the eating area prevented mice from hoarding food. (B) Mean weight of mice (n=33) during the three weeks of the experiment; for the first two weeks, the motorized cover was left open to allow mice to get acclimated to the cage. At days 15 and 16, the cover opened (with a noise) for 11 minutes every three hours, which left the mice hungry and got them used to feed every time the cover opened. From day 17 to time of sacrifice (day 21), the cover opened for 16 min every three hours, which allowed mice to regain normal weight. (C) Mean weights (n=33) and standard deviations.
