# Supplementary material for: Diurnal regulation of RNA polymerase III transcription is under the control of both the feeding–fasting response and the circadian clock
Source: Genome Res. 2017 Jun;27(6):973–84. doi: 10.1101/gr.217521.116 (PMC5453330; doi:10.1101/gr.217521.116)
Supplement: Supplemental Material [file supp_gr.217521.116_Supplemental_Fig_S2.pdf]

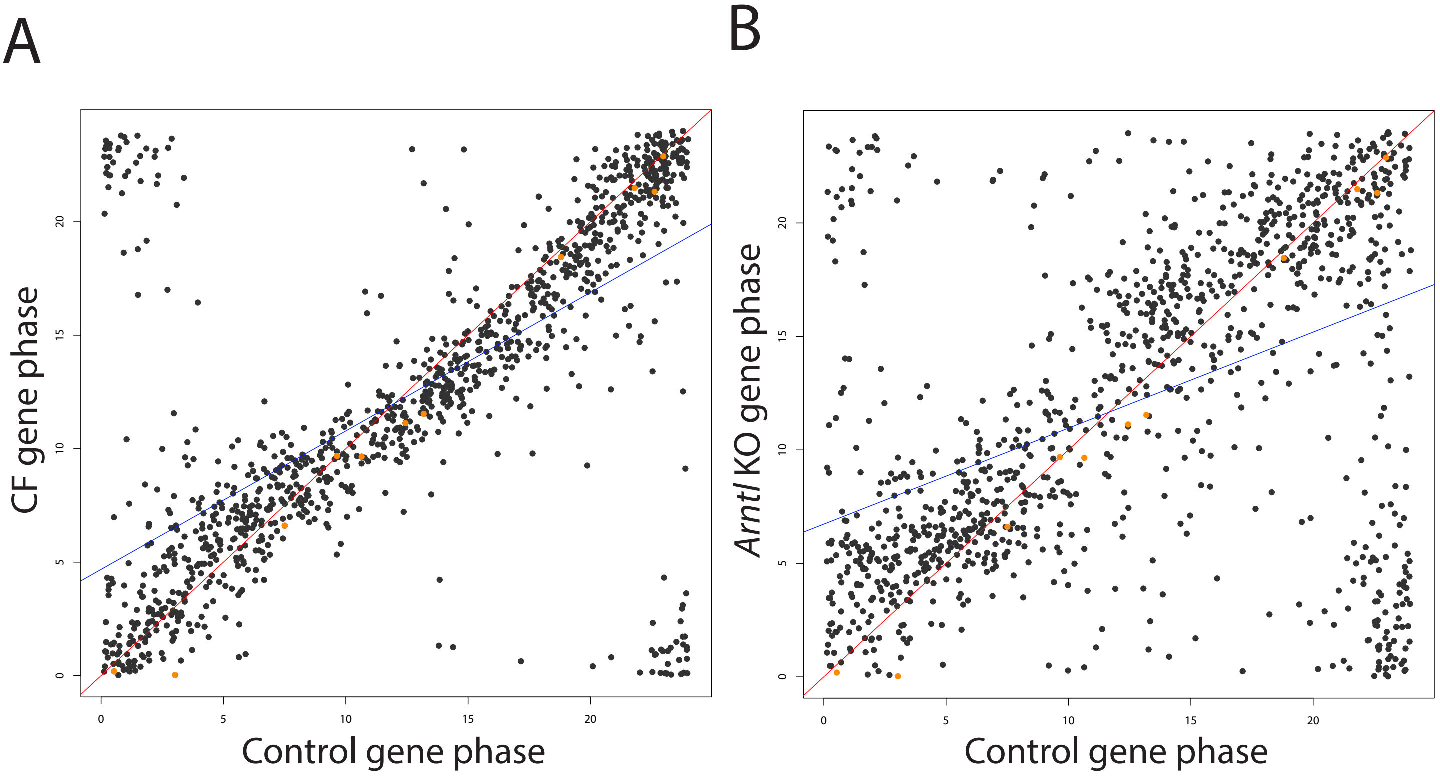

Supplemental Fig. S2. Phase analysis.

(A) Scatter plots showing phases for the genes in the control dataset in control (x-axis) and CF (y-axis) liver. The red line is the X = Y line and the blue line is the best fit. The orange dots are circadian-related genes, selected according to gene ontology analysis. (B) As in A but in control (x-axis) and Arntl KO (y-axis) liver.
