# Supplementary material for: Diurnal regulation of RNA polymerase III transcription is under the control of both the feeding–fasting response and the circadian clock
Source: Genome Res. 2017 Jun;27(6):973–84. doi: 10.1101/gr.217521.116 (PMC5453330; doi:10.1101/gr.217521.116)
Supplement: Supplemental Material [file supp_gr.217521.116_Supplemental_Fig_S3.pdf]

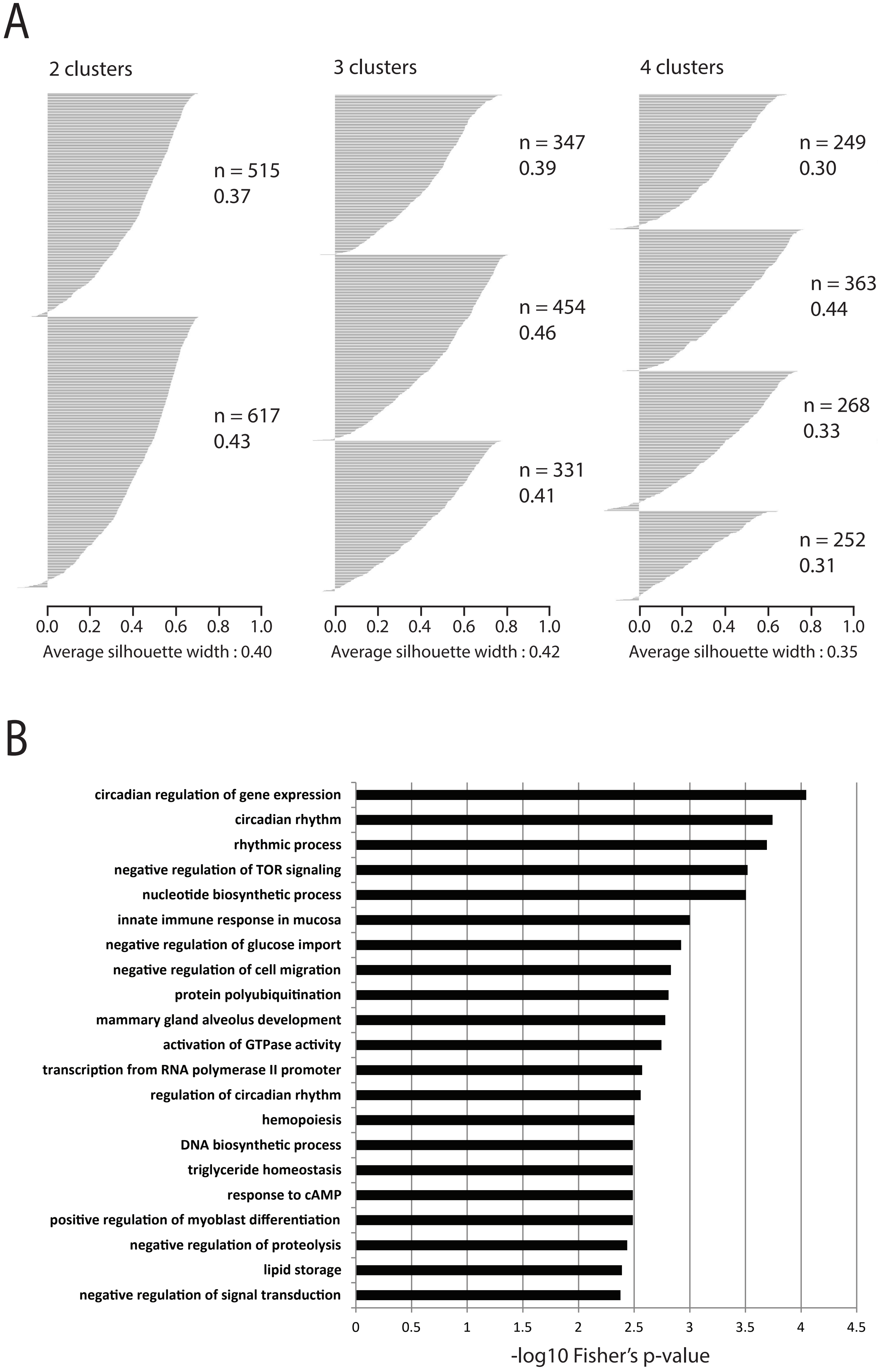
A
2 clusters
n = 515
0.37
n = 617
0.43
0.0 0.2 0.4 0.6 0.8 1.0
Average silhouette width : 0.40
3 clusters
n = 347
0.39
n = 454
0.46
n = 331
0.41
0.0 0.2 0.4 0.6 0.8 1.0
Average silhouette width : 0.42
4 clusters
n = 249
0.30
n = 363
0.44
n = 268
0.33
n = 252
0.31
0.0 0.2 0.4 0.6 0.8 1.0
Average silhouette width : 0.35
B
circadian regulation of gene expression
circadian rhythm
rhythmic process
negative regulation of TOR signaling
nucleotide biosynthetic process
innate immune response in mucosa
negative regulation of glucose import
negative regulation of cell migration
protein polyubiquitination
mammary gland alveolus development
activation of GTPase activity
transcription from RNA polymerase II promoter
regulation of circadian rhythm
hemopoiesis
DNA biosynthetic process
triglyceride homeostasis
response to cAMP
positive regulation of myoblast differentiation
negative regulation of proteolysis
lipid storage
negative regulation of signal transduction
0 0.5 1 1.5 2 2.5 3 3.5 4 4.5
-log10 Fisher's p-value

C

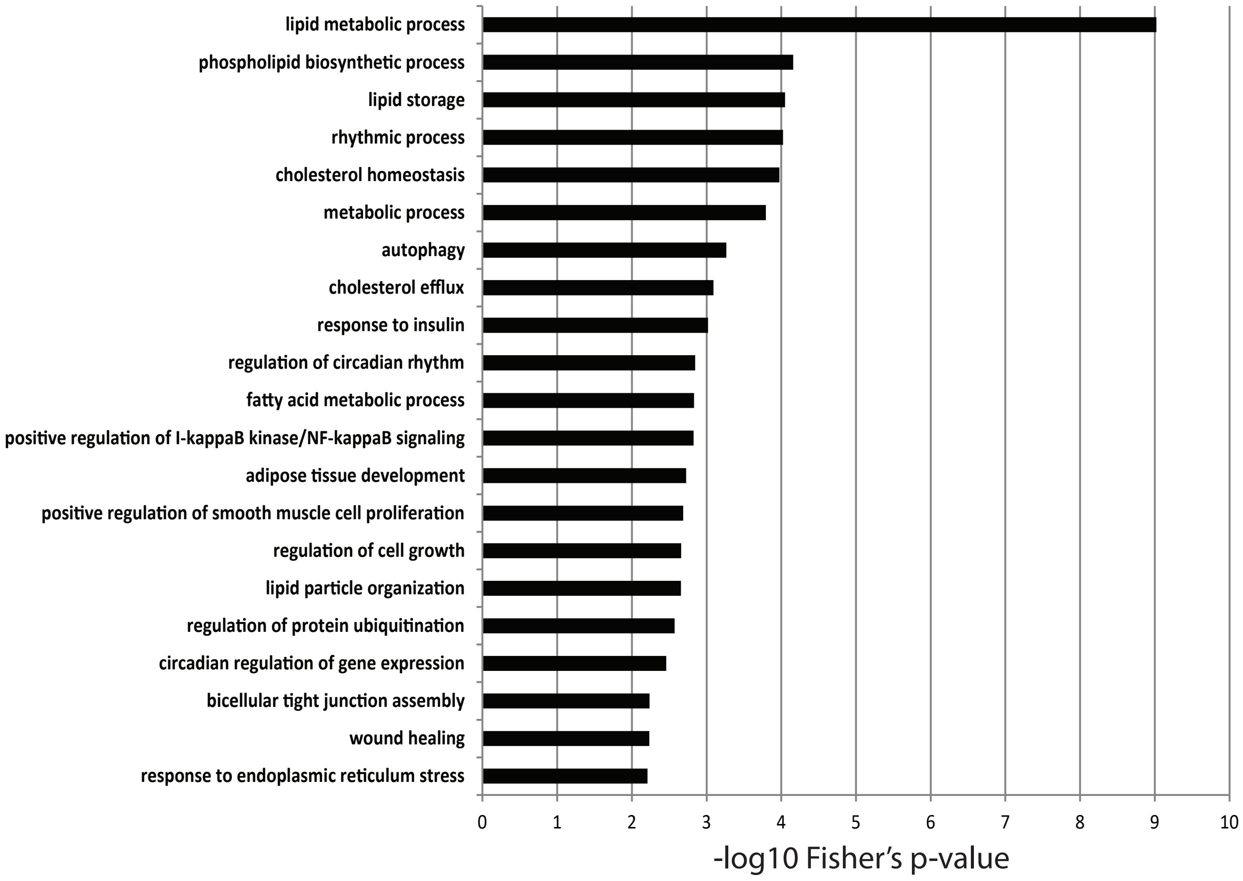
lipid metabolic process
phospholipid biosynthetic process
lipid storage
rhythmic process
cholesterol homeostasis
metabolic process
autophagy
cholesterol efflux
response to insulin
regulation of circadian rhythm
fatty acid metabolic process
positive regulation of I-kappaB kinase/NF-kappaB signaling
adipose tissue development
positive regulation of smooth muscle cell proliferation
regulation of cell growth
lipid particle organization
regulation of protein ubiquitination
circadian regulation of gene expression
bicellular tight junction assembly
wound healing
response to endoplasmic reticulum stress
0 1 2 3 4 5 6 7 8 9 10
-log10 Fisher's p-value

D

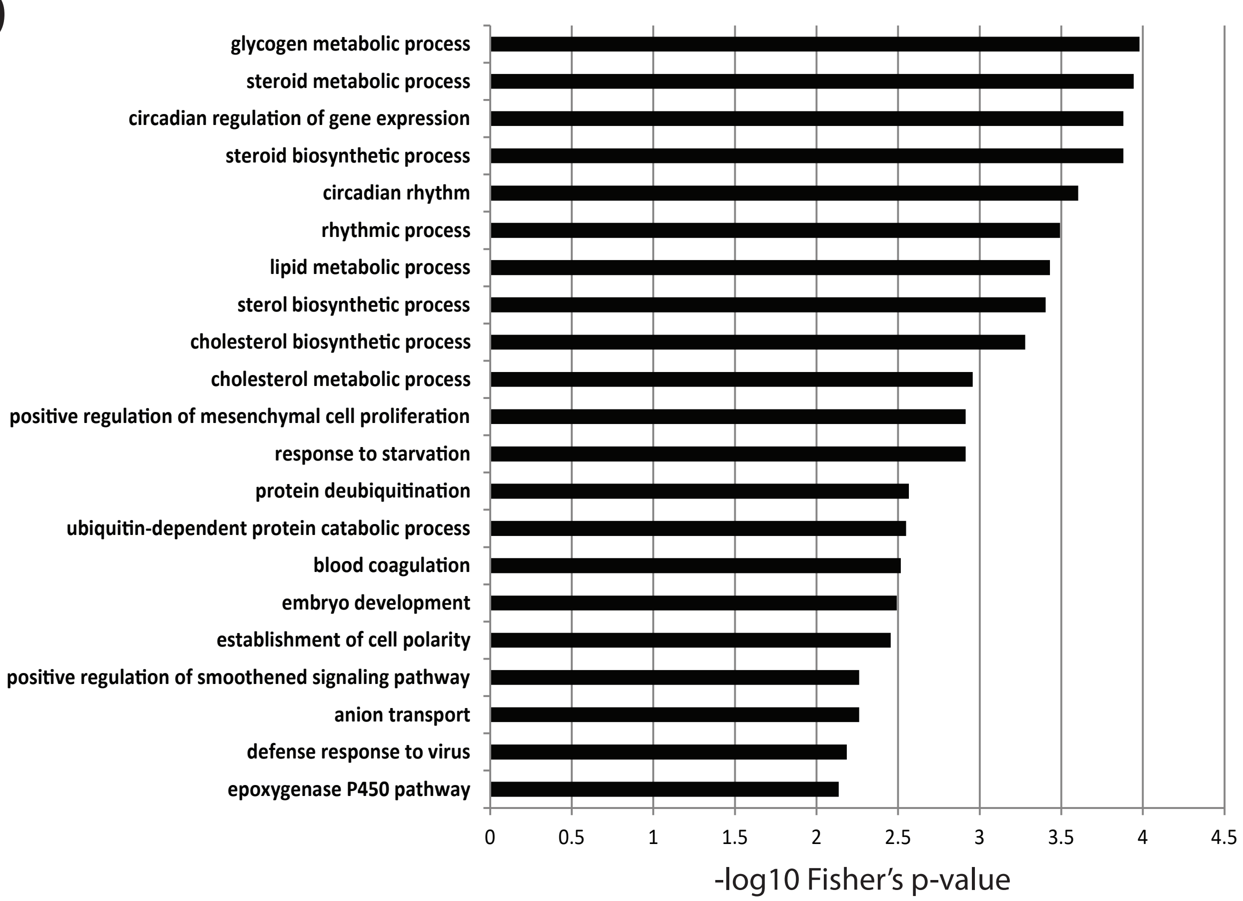
glycogen metabolic process
steroid metabolic process
circadian regulation of gene expression
steroid biosynthetic process
circadian rhythm
rhythmic process
lipid metabolic process
sterol biosynthetic process
cholesterol biosynthetic process
cholesterol metabolic process
positive regulation of mesenchymal cell proliferation
response to starvation
protein deubiquitination
ubiquitin-dependent protein catabolic process
blood coagulation
embryo development
establishment of cell polarity
positive regulation of smoothened signaling pathway
anion transport
defense response to virus
epoxygenase P450 pathway
0 0.5 1 1.5 2 2.5 3 3.5 4 4.5
-log10 Fisher's p-value

Supplemental Fig. S3. Gene ontology analysis for the three clusters in the PAM analysis.

(A) Silhouette analysis of clustering into 2, 3, or 4 clusters for the control rhythmic dataset in the control condition. Each bar represents the silhouette width for a given gene. The number of genes and the average width for each cluster are indicated on the right of each cluster. Clustering into three clusters was chosen for the PAM analysis shown in Figures 2D and S4D. (B) Top 20 GO terms associated with the 347 genes in cluster 1. (C) As in B but for the 454 genes in the cluster 2. (D) As in B but for the 331 genes in the cluster 3. Bars represent the –log10 of the p-value.
