# Supplementary figures and images for: Diurnal regulation of RNA polymerase III transcription is under the control of both the feeding–fasting response and the circadian clock
Source: Genome Res. 2017 Jun;27(6):973–84. doi: 10.1101/gr.217521.116 (PMC5453330; doi:10.1101/gr.217521.116)

A

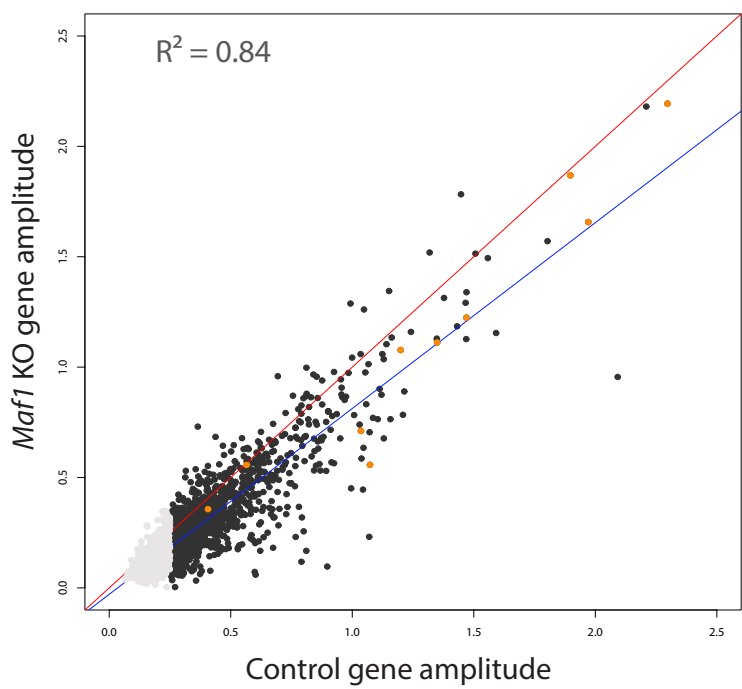

B

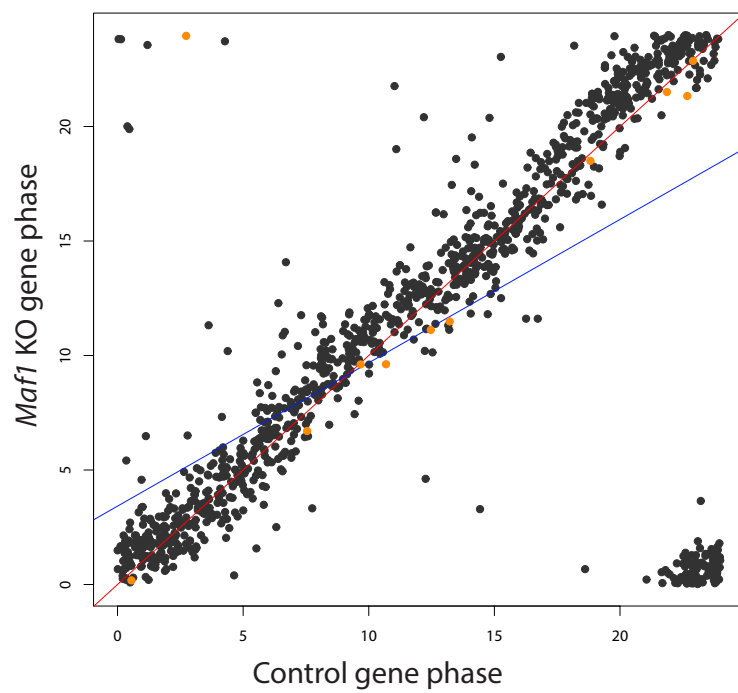

C

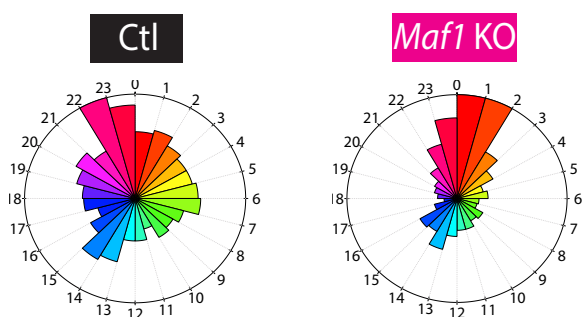

D

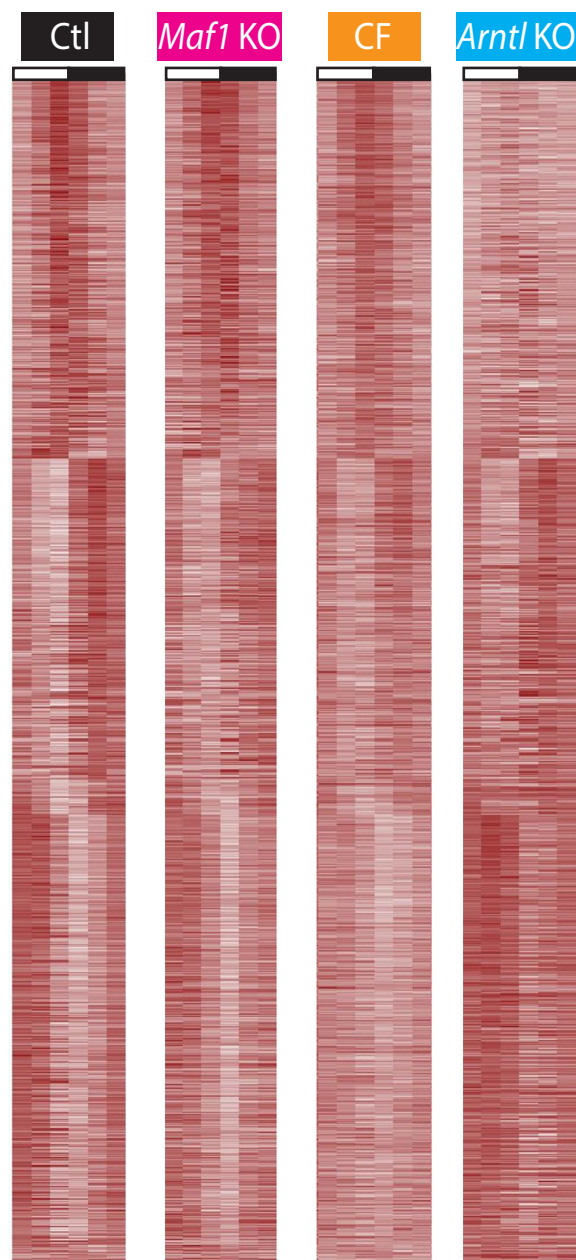

E

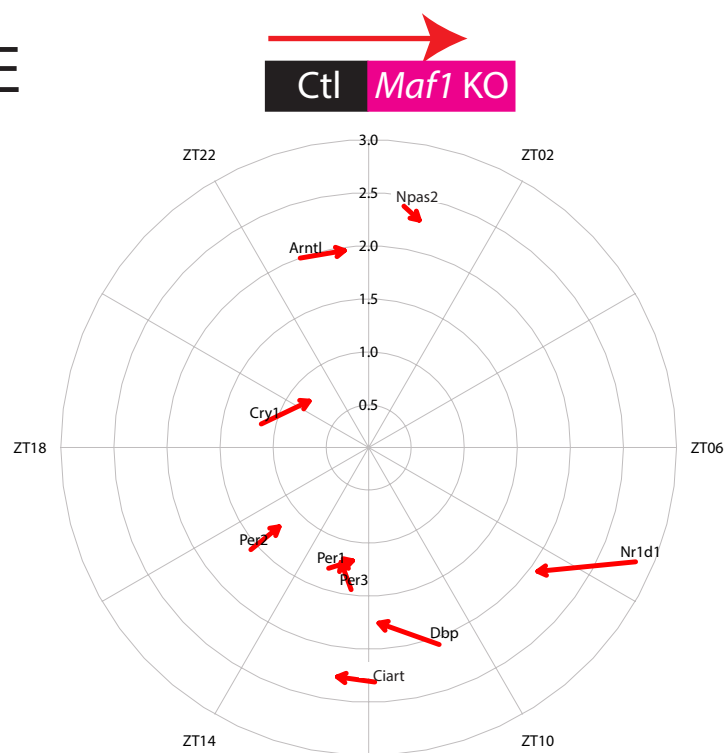

Supplement: Supplemental Material [file supp_gr.217521.116_Supplemental_Fig_S4.pdf]
